# NOX2 deficiency alters macrophage phenotype through an IL-10/STAT3 dependent mechanism: implications for traumatic brain injury

**DOI:** 10.1186/s12974-017-0843-4

**Published:** 2017-03-24

**Authors:** James P. Barrett, Rebecca J. Henry, Sonia Villapol, Bogdan A. Stoica, Alok Kumar, Mark P. Burns, Alan I. Faden, David J. Loane

**Affiliations:** 1 Department of Anesthesiology and Shock, Trauma and Anesthesiology Research (STAR) Center, University of Maryland School of Medicine, 655 West Baltimore Street, #6-011, Baltimore, MD 21201 USA; 2 Laboratory for Brain Injury and Dementia, Department of Neuroscience, Georgetown University, Washington, DC USA

**Keywords:** NADPH oxidase, NOX2, Traumatic brain injury, Macrophage, Neuroinflammation, Interleukin-10

## Abstract

**Background:**

NADPH oxidase (NOX2) is an enzyme system that generates reactive oxygen species (ROS) in microglia and macrophages. Excessive ROS production is linked with neuroinflammation and chronic neurodegeneration following traumatic brain injury (TBI). Redox signaling regulates macrophage/microglial phenotypic responses (pro-inflammatory versus anti-inflammatory), and NOX2 inhibition following moderate-to-severe TBI markedly reduces pro-inflammatory activation of macrophages/microglia resulting in concomitant increases in anti-inflammatory responses. Here, we report the signaling pathways that regulate NOX2-dependent macrophage/microglial phenotype switching in the TBI brain.

**Methods:**

Bone marrow-derived macrophages (BMDMs) prepared from wildtype (C57Bl/6) and NOX2 deficient (NOX2^−/−^) mice were treated with lipopolysaccharide (LPS; 10 ng/ml), interleukin-4 (IL-4; 10 ng/ml), or combined LPS/IL-4 to investigate signal transduction pathways associated with macrophage activation using western immunoblotting and qPCR analyses. Signaling pathways and activation markers were evaluated in ipsilateral cortical tissue obtained from adult male wildtype and NOX2^−/−^ mice that received moderate-level controlled cortical impact (CCI). A neutralizing anti-IL-10 approach was used to determine the effects of IL-10 on NOX2-dependent transitions from pro- to anti-inflammatory activation states.

**Results:**

Using an LPS/IL-4-stimulated BMDM model that mimics the mixed pro- and anti-inflammatory responses observed in the injured cortex, we show that NOX2^−/−^ significantly reduces STAT1 signaling and markers of pro-inflammatory activation. In addition, NOX2^−/−^ BMDMs significantly increase anti-inflammatory marker expression; IL-10-mediated STAT3 signaling, but not STAT6 signaling, appears to be critical in regulating this anti-inflammatory response. Following moderate-level CCI, IL-10 is significantly increased in microglia/macrophages in the injured cortex of NOX2^−/−^ mice. These changes are associated with increased STAT3 activation, but not STAT6 activation, and a robust anti-inflammatory response. Neutralization of IL-10 in NOX2^−/−^ BMDMs or CCI mice blocks STAT3 activation and the anti-inflammatory response, thereby demonstrating a critical role for IL-10 in regulating NOX2-dependent transitions between pro- and anti-inflammatory activation states.

**Conclusions:**

These studies indicate that following TBI NOX2 inhibition promotes a robust anti-inflammatory response in macrophages/microglia that is mediated by the IL-10/STAT3 signaling pathway. Thus, therapeutic interventions that inhibit macrophage/microglial NOX2 activity may improve TBI outcomes by not only limiting pro-inflammatory neurotoxic responses, but also enhancing IL-10-mediated anti-inflammatory responses that are neuroprotective.

**Electronic supplementary material:**

The online version of this article (doi:10.1186/s12974-017-0843-4) contains supplementary material, which is available to authorized users.

## Background

Macrophages play a critical role in regulating the innate immune response to acute and chronic inflammation, and they can change their activation state in response to growth factors and external cues [1]. In the central nervous system (CNS) following traumatic brain injury (TBI), resident microglia and infiltrating macrophages respond to the local injury; depending upon their activation state, they may contribute either to secondary injury or neurorestoration [2]. Macrophages exhibit a diverse array of activation phenotypes, each with different physiological and functional roles [1, 3, 4]. In vitro studies have demonstrated that pro-inflammatory (M1-like) activation is characterized by upregulation of inflammatory mediators such as nitric oxide synthase (NOS2) and pro-inflammatory cytokines (tumor necrosis factor α (TNFα), interleukin-1β (IL-1β)) and is associated with production of reactive oxygen species (ROS) [1]. It can be induced by the pro-inflammatory cytokine interferon (IFN)-γ and activation of toll-like receptors (TLR). IFN-γ stimulation leads to phosphorylation of the transcription factor signal transducer activator of transcription (STAT)1, and effects of the TLR4 agonist, lipopolysaccharide (LPS), are mediated, in part, through STAT1 [5]. In contrast, anti-inflammatory (M2-like) macrophages are involved in both wound healing and resolution of inflammatory responses [4]. They are marked by expression of factors such as mannose receptor (CD206), arginase-1 (Arg1), and chitinase 3-like 3 (YM1). Stimulation of macrophages with interleukin-4 (IL-4) upregulates the expression of markers of the anti-inflammatory phenotype—effects mediated in part through activation of STAT6 [6]. Other inflammatory mediators such as interleukin-10 (IL-10) [7] and insulin-stimulating growth factor-1 (IGF-1) [8] may also activate the anti-inflammatory phenotype.

Defining specific activation states in vivo is challenging due to the diversity of phenotypes, which are dependent on environmental and tissue-specific cues that allow microglia/macrophages to perform specific functions under different physiological and pathological conditions. The in vitro-defined phenotypic classification system (M1 versus M2 polarization) oversimplifies the activation spectrum that exists in humans and animal models [9] and does not describe the actual diversity of microglial/macrophage reactive states. This has led to serious questioning of the use of M1/M2 classification in microglia [10] and peripheral macrophages [11]. The actual mechanisms underlying phenotypic changes in microglia/macrophages are not fully elucidated, and an accepted nomenclature that describes such complex cellular responses is currently lacking. It has been demonstrated that the microglial/macrophage activation response to TBI is highly complex, with individual cells concurrently expressing both pro- and anti-inflammatory phenotype markers in unique profiles that can be altered as a function of time, injury localization, and injury severity [12–14]. In the current study, we use general terms, pro-inflammatory, and anti-inflammatory, to describe microglial/macrophage phenotypic responses in the TBI brain.

NADPH oxidase (NOX2) is a multi-subunit enzyme complex responsible for the production of both intracellular and extracellular ROS by phagocytes, including microglia and macrophages [15]. NOX2 is the primary source of ROS generation in macrophages [16], and it participates in a wide range of cellular processes, including host defense, cell signaling, and cell differentiation. Excessive NOX2 activation is implicated in the progression of a number of neurodegenerative disorders [17–19]. Following a moderate-to-severe TBI NOX2 is chronically elevated in reactive macrophages/microglia surrounding the lesion for weeks to months post-injury [20, 21]. NOX2 expression is also markedly increased in aged animals after TBI and is associated with increased pro-inflammatory activation and suppressed anti-inflammatory marker expression [22]. Notably, NOX2 inhibition reduces markers of pro-inflammatory activation, limits tissue loss and neurodegeneration, and improves neurological recovery in TBI mice [12, 23–26]. NOX2 inhibition also results in enhanced expression of anti-inflammatory markers in macrophage/microglia [24, 27], and infiltrating macrophages are major contributors to the anti-inflammatory environment in the TBI brain in NOX2 knockout mice [24]. Based on accumulating pre-clinical evidence supporting NOX2-mediated neuroinflammation as an important therapeutic target for TBI, the goal of the current study was to elucidate the cell signaling mechanisms that regulate NOX2-dependent phenotypic changes in macrophages/microglia in the brain following TBI.

## Methods

### Animals

Studies were performed using either NOX2-deficient (NOX2^−/−^; B6.129S-Cybb^tm1Din^/J, stock 002365; Jackson Laboratories, Bar Harbor, ME,) adult male mice (10–12 weeks old), or age-matched C57Bl/6 J (WT) male mice. Mice were housed in the Animal Care facility at the University of Maryland School of Medicine under a 12 h light–dark cycle, with ad libitum access to food and water. All surgical procedures were carried out in accordance with protocols approved by the Institutional Animal Care and Use Committee (IACUC) at the University of Maryland School of Medicine.

### Controlled cortical impact

Our custom-designed controlled cortical impact (CCI) injury device consists of a microprocessor-controlled pneumatic impactor with a 3.5-mm diameter tip as previously described [28]. Briefly, mice were anesthetized with isoflurane evaporated in a gas mixture containing 70% N_2_O and 30% O_2_ and administered through a nose mask. Mice were placed on a heated pad, and core body temperature was maintained at 37 °C. The head was mounted in a stereotaxic frame, a 10-mm midline incision was made over the skull, and the skin and fascia were reflected. A 5-mm craniotomy was made on the central aspect of the left parietal bone. The impounder tip of the injury device was then extended to its full stroke distance (44 mm), positioned to the surface of the exposed dura, and reset to impact the cortical surface. Moderate-level CCI was induced using an impactor velocity of 6 m/s and deformation depth of 2 mm. After injury, the incision was closed with interrupted 6–0 silk sutures, anesthesia was terminated, and the animal was placed into a heated cage to maintain normal core temperature for 45 min post-injury. Sham animals underwent the same procedure as CCI mice except for the impact.

### Intracerebroventricular (i.c.v.) guide cannula implantation and osmotic pump infusion

Prior to CCI, the right lateral ventricle of the mouse was stereotaxically perforated with a brain infusion kit 3 (ALZET, DURET Corporation, Cupertino, CA, USA; coordinates: 0.7 mm posterior to the bregma, 1.5 mm lateral to the bregma, 2 mm deep). Immediately following CCI on the left parietal cortex the infusion cannula was connected to an osmotic minipump (ALZET; pump model: 1007D) that was implanted subcutaneously (sc) in the animal’s back, just behind scapula. Osmotic pumps were primed for approximately 8 h prior to implantation and were either filled with 1 mg/ml anti-IL-10 neutralizing antibody (αIL-10; clone JES5-16E3; eBioscience, San Diego, CA) or equal concentration isotype control rat αIgG2b K (clone Eb149/10h5 eBioscience). Once implanted, the pumps continually infused αIL-10 or control αIgG2b K into the lateral ventricle for 3 days at a rate of 0.5 μL/h.

### Study 1

Sham (*n* = 5) and CCI (*n* = 5) of WT and NOX2^−/−^ mice were anesthetized (100 mg/kg sodium pentobarbital, I.P.) at 1, 3, and 7 days post-injury and transcardially perfused with ice-cold 0.9% saline (100 ml). Ipsilateral cortical tissue was rapidly dissected and was snap-frozen on liquid nitrogen for RNA extraction.

### Study 2

Sham (*n* = 5) and CCI (*n* = 5) of WT and NOX2^−/−^ mice were anesthetized (100 mg/kg sodium pentobarbital, I.P.) at 7 days post-injury and transcardially perfused with ice-cold 0.9% saline (100 ml), followed by 300 ml of 4% paraformaldehyde. Brains were removed and post-fixed in 4% paraformaldehyde overnight and were cryoprotected in 30% sucrose for histological analysis.

### Study 3

αIL-10 (1 mg/ml) or isotype control αIgG2b K was delivered i.c.v. via osmotic pump infusion to WT and NOX2^−/−^ CCI mice (*n* = 6/group). The dose of αIL-10 was chosen based on prior studies demonstrating neutralization of IL-10 in a mouse model of spinal cord injury (SCI) [29]. Sham WT mice (*n* = 6) were used as control for baseline mRNA expression levels. Animals were anesthetized (100 mg/kg sodium pentobarbital, I.P.) at 3 days post-injury and transcardially perfused with ice-cold 0.9% saline (100 ml). Ipsilateral cortical tissue was rapidly dissected and was snap-frozen on liquid nitrogen for RNA extraction.

### Preparation and culture of bone marrow-derived macrophages (BMDMs)

BMDMs were isolated from the marrow of the femurs and tibias of uninjured adult WT and NOX2^−/−^ male mice as previously described [8]. Briefly, following terminal anesthesia (100 mg/kg sodium pentobarbital, I.P.), the marrow was flushed out into a sterile falcon tube in Dulbecco’s modified Eagle’s medium (DMEM; Gibco Invitrogen, Carlsbad, CA) supplemented with heat-inactivated fetal bovine serum (FBS; 10%; Atlanta Biologicals, Flowery Branch, GA) and penicillin/streptomycin (1%; Gibco Invitrogen) to obtain a single cell suspension. The suspension was centrifuged (400 × *g*, 5 min), the supernatant was discarded, and the resulting pellet was resuspended in 20 ml of DMEM supplemented with Ladmac-conditioned media (20%). Cells were seeded in sterile cell culture flasks (T75 cm^2^ flasks) and were maintained in culture for a further 6 days, with media being replaced on day 4. On day 6, cells were transferred to 24-well plates (0.4 × 10^6^ cells per well) or 96-well plates (0.5 × 10^5^ cells per well) and remained in culture for a further 2 days.

### Treatment of BMDMs

Cells were incubated in the presence of both LPS (10 ng/ml; Sigma-Alrich, St. Louis, MO) and IL-4 (10 ng/ml; R&D Systems, Minneapolis, MN), supernatants were collected for analysis of cytokines by ELISA, and cells were harvested for analysis of markers of macrophage activation by real-time PCR and polyacrylamide gel electrophoresis followed by western immunoblotting. To investigate IL-10-mediated effects in anti-inflammatory activation, cells were treated with LPS/IL-4 in the presence or absence of a neutralizing antibody to αIL-10 (10 μg/ml; eBioscience) or the appropriate isotype control (rat αIgG2b K; eBioscience).

### Real-time PCR

Total RNA was extracted from BMDMs and snap-frozen sham and TBI cortical tissue of WT and NOX2^−/−^ mice using an RNeasy isolation kit (Qiagen, Valencia, CA) with on-column DNase treatment (Qiagen). cDNA synthesis was performed using a Verso cDNA RT kit (Thermo Scientific, Pittsburg, PA); the protocols used were according to the manufacturer’s instructions. Real-time PCR was performed using TaqMan gene expression assays (Ym1, Mm00657889_m1; Arg1, Mm00475988_m1; SOCS3, Mm00545913_s1; IL-4Rα, Mm00446186_m1; TGF-β, Mm00441724_m1; SHIP1, Mm00494987_m1; and GAPDH Mm99999915_g1; Applied Biosystems, Carlsbad, CA) on an ABI 7900 HT FAST Real-Time PCR machine (Applied Biosystems). Samples were assayed in duplicate in one run (40 cycles), which was composed of 3 stages, 50 °C for 2 min, 95 °C for 10 s for each cycle (denaturation) and finally the transcription step at 60 °C for 1 min. Gene expression was calculated relative to the endogenous control sample (GAPDH) to determine relative expression values, using the 2 − ΔΔCt method (where Ct is the threshold cycle).

### Western blotting

Proteins from BMDMs and ipsilateral cortical tissue were extracted using RIPA buffer, equalized, and loaded onto 5–20% gradient gels for SDS PAGE (Bio-Rad; Hercules, CA). Proteins were transferred onto nitrocellulose membranes and then blocked for 1 h in 5% milk in 1 × TBS containing 0.05% Tween-20 (TBS-T) at room temperature. The membrane was incubated in mouse anti-arginase 1 (N-20) (1:1000; BD Transduction Laboratories, San Jose, CA), rabbit anti-STAT6 (1:1000; Cell signaling, Danvers, MA), rabbit anti-pSTAT6 (1:1000; Cell signaling), mouse anti-STAT3 (1:1000; Cell signaling), rabbit anti-pSTAT3 (1:1000; Cell signaling), or mouse anti-β-Actin (1:5000; Sigma-Aldrich) overnight at 4 °C, then washed three times in TBS-T, and incubated in appropriate HRP-conjugated secondary antibodies (Jackson ImmunoResearch Laboratories, West Grove, PA) for 2 h at room temperature. Membranes were washed three times in TBS-T, and proteins were visualized using SuperSignal West Dura Extended Duration Substrate (Thermo Scientific, Rockford, IL). Chemiluminescence was captured ChemiDoc™ XRS+ System (Bio-Rad), and protein bands were quantified by densitometric analysis using BioRad Molecular Imaging Software. The data presented reflects the intensity of target protein band normalized based on the intensity of the endogenous control for each sample (expressed in arbitrary units).

### Cytokine and nitric oxide analysis

Concentrations of TNFα (R&D Systems; DY410), IL-6 (R&D Systems; DY406), and IL-10 (R&D Systems; DY41705) were measured in supernatant samples obtained from BMDMs by ELISA. Briefly, standards or samples (100 μl) were added to antibody-coated 96-well plates and incubated for 2 h at room temperature, plates were washed and samples were incubated in detection antibody for 2 h. Plates were washed and incubated in horseradish peroxidase-conjugated streptavidin for 20 min at room temperature. Substrate solution (tetramethylbenzidine; Sigma-Alrich) was added, incubation continued at room temperature in the dark for 30 min, and the reaction was stopped using H_2_SO_4_ (1 M). Absorbance measurements were read at 450 nm using a Synergy HT Multi-Mode Microplate Reader (Biotek, Winooski, VT). Cytokine concentrations were calculated relative to the appropriate standard curve and expressed as pg cytokine/μg of protein. Nitric oxide (NO) release was assayed using a Greiss reagent assay (Invitrogen; G7921), per the manufacturer’s instructions. NO concentrations were calculated using standard curves generated from a nitrite stock, and results were expressed in micromoles.

### IL-10 fluorescent in situ hybridization (FISH), immunohistochemistry, and quantification of IL-10 co-localization

20 μm coronal brain sections were cut on a cryostat, mounted on gelatin-coated glass slides (Superfrost Plus, Fisher Scientific, Tusin, CA), and stored at −80 °C until use. Fluorescent in situ hybridization (FISH) was performed as per the manufacturer’s instructions using RNAscope® Technology 2.0 Red Fluorescent kit for Fresh Frozen Tissue (Advanced Cell Diagnostics, Inc., Hayward, CA). Tissue sections were dehydrated by 50, 70, and 100% ethanol gradually for 5 min, and then boiled for 3–5 min with pretreatment 2 solution, incubated with pretreatment 3 solution, and incubated with target IL-10 probe (Mus musculus interleukin 10 (IL-10) mRNA; accession number NM_010548.2, target region 2–1095) at 40 °C for 2 h in the HybEZ humidified incubator. A dapB probe targeting a bacterial gene was used as a negative control and Ppib (Mus musculus peptidylprolyl isomerase B mRNA; accession number NM_011149.2, target region 98–856) was used as a positive control. After FISH, slides were washed three times with PBS and blocking with PBS containing 0.5% Triton X-100 and 2% normal goat serum for 1 h. Immunohistochemistry was performed using polyclonal anti-rabbit Iba-1 (1:500, Wako Chemicals, Richmond, VA) incubated overnight at 4 °C. Alexa Fluor 488-conjugated goat anti-rabbit IgG (1:1000, Invitrogen, Carlsbad, CA) was applied for 1 h at room temperature. Sections were rinsed with PBS three times and incubated in PBS with DAPI solution (1:50.000) for counterstained nuclei. The sections were washed three times in distilled water and coverslipped with Fluoro-Gel with Tris Buffer mounting medium (Electron Microscopy Sciences, Hatfield, PA). Experiments were repeated three times.

All analyses were performed on digital images captured using a ×20 objective in a sampled region within the ipsilateral primary somatosensory cortex with a field of 151.894 mm^2^ around the impact site. Two to three fields were randomly selected per brain section (see Fig 6 B1) between −1.70 and −2.30 mm from the bregma, and a total of 2 to 4 brain sections per brain. Images were taken in the cortical layers III–IV for sham mice. IL-10 positive cells (red) that colocalized with DAPI nuclei (blue) were manually counted. The negative control probe did not contain any stained cells. Stained cells were examined under a florescent microscope (Axioplan 2 Zeiss) and were analyzed using Image J software (http://rsbweb.nih.gov/ij/). RNA positive cells were quantitated in each cortical sampling zone, each field of view analyzed was the sum of three images of sequential optical Z-stack images combined into a single overlay image, and IL-10 positive cells were counted using the overlay. The result from all the fields of view in a given animal was averaged to obtain the value for that individual (*n* = 5/group). Double positive cells were identified using the IL-10 probe and colocalization with Iba-1 (green) were based on analysis of microscopic fields of view as previously described [30], keeping all acquisition parameters constant. The percentage of mRNA IL-10 positive cells that colocalized with Iba-1 immunoreactive cells relative to the total number of IL-10 positive cells was assessed. A confocal microscope (Leica SP8) was used to take images from FISH-ISH brain sections using ×20, ×40, and ×63 objectives.

### Statistical analysis

Randomization protocols were employed in all experiments, and individuals performing analysis were blinded to treatment and genotype groups. Data are reported as the mean ± SEM, and the number of experiments is indicated in each case. Statistical analysis was carried out using a two- or three-way analysis of variance (ANOVA) with post hoc Bonferroni tests or one-way ANOVA followed by post hoc Newman-Keuls analysis to identify specific differences between groups. When comparisons were being made between two conditions, an unpaired Student’s *t* test was performed. Significance level was set as *P* < 0.05.

## Results

### LPS/IL-4 induces a mixed phenotype in BMDMs

**Fig. 1:**
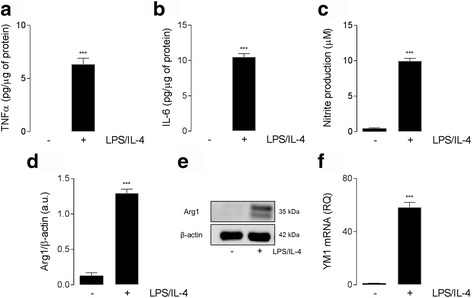
LPS/IL-4 induces a mixed phenotype in BMDMs. (**a-f**) LPS/IL-4 (both 10 ng/ml; 24 h) stimulation significantly increased the production of TNFα (**a**), IL-6 (**b**), and Nitrite (**b**) in BMDMs from WT mice (^***^ *P*< 0.001; Student’s *t* test). LPS/IL-4 increased Arg1 (**d**) protein expression (representative western immunoblot shown (**e**)) and YM1 (**f**) mRNA expression in BMDMs (^***^ *P* < 0.001; Student’s *t* test). All data are expressed as means (±SEM; *n* = 3)

It is well documented that macrophages/microglia adopt distinct phenotypes in response to inflammatory stimuli such as LPS (pro-inflammatory) or IL-4 (anti-inflammatory) [31, 32], however, recently it has been observed that after TBI, macrophages/microglia express markers associated with both pro- and anti-inflammatory phenotypes [12–14]. To mimic this activation spectrum in vitro, BMDMs were incubated in the presence of both LPS (10 ng/ml) and IL-4 (10 ng/ml), and this combined treatment significantly upregulated both pro- and anti-inflammatory activation markers. Specifically, LPS/IL-4 stimulation led to a significant increase in the supernatant concentration of TNFα (*P* < 0.001; Student’s *t* test; Fig. 1a) and IL-6 (*P* < 0.001; Student’s *t* test; Fig. 1b), both of which are cytokines associated with a pro-inflammatory phenotype. We also observed elevated levels of nitrite (*P* < 0.001; Student’s *t* test; Fig. 1c), an indication of increased NO production. The changes in pro-inflammatory markers were also accompanied by an increase in Arg1 protein (*P* < 0.001; Student’s *t* test; Fig. 1d), and an upregulation in YM1 mRNA expression (*P* < 0.001; Student’s *t* test; Fig. 1f), both markers of anti-inflammatory macrophages. Of note, when we stimulated BMDMs with only LPS or IL-4 alone, there was increased expression of markers of pro- and anti-inflammatory phenotypes, respectively, but mixed pro- and anti-inflammatory phenotype markers were not detected (data not shown). These results demonstrate that LPS/IL-4 stimulation of BMDMs produces a mixed pro- and anti-inflammatory activation state, similar to the activation profile observed in the TBI brain.

### NOX2 deficiency alters macrophage response to LPS/IL-4

**Fig. 2:**
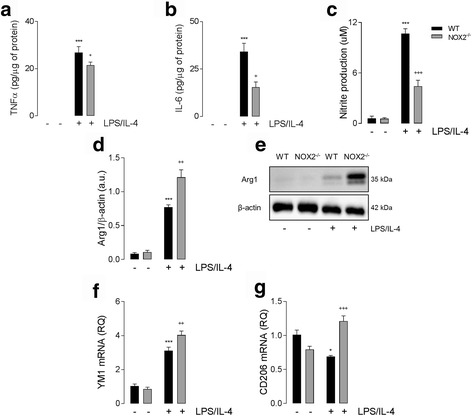
NOX2 deficiency alters macrophage response to LPS/IL-4. LPS/IL-4 (both 10 ng/ml; 24 h) stimulation significantly increased the release of TNFα (**a**), IL-6 (**b**), and Nitrite (**c**) in WT and NOX2^−/−^ BMDMs (^***^ *P* < 0.001; ANOVA). LPS/IL-4-induced increase in pro-inflammatory markers was significantly reduced in and NOX2^−/−^ BMDMs compared with WT BMDMs (^++^ *P* < 0.01, ^+++^ *P* < 0.001, WT LPS/IL-4 vs. NOX2^−/−^ LPS/IL-4; ANOVA). LPS/IL-4 increased Arg1 (**d**) protein expression (representative western immunoblot shown (**e**)) and YM1 (**f**) mRNA expression in WT and NOX2^−/−^ (^***^ *P* < 0.001; ANOVA). Expression of anti-inflammatory markers was significantly increased in NOX2^−/−^ BMDMs compared with WT BMDMs (^++^ *P* < 0.01, ^+++^ *P* < 0.001, WT LPS/IL-4 vs. NOX2^−/−^ LPS/IL-4; ANOVA). LPS/IL-4 significantly decreased CD206 mRNA expression in WT BMDMs (^*^ *P* < 0.05; ANOVA; **g**), while CD206 mRNA expression was significantly increased in NOX2^−/−^ BMDMs following LPS/IL-4 stimulation (^+++^ *P* < 0.001, NOX2^−/−^ con vs. NOX2^−/−^ LPS/IL-4; ANOVA). All data are expressed as means (±SEM; *n* = 6)

Given that NOX2^−/−^ TBI mice exhibit enhanced anti-inflammatory activation that is associated with improved outcomes following TBI [24], we set out to investigate mechanisms of NOX2-dependent macrophage phenotype switching using the LPS/IL-4 stimulation model. WT and NOX2^−/−^ BMDMs were treated with LPS/IL-4 for 24 h, and pro and anti-inflammatory activation markers were assessed. LPS/IL-4 significantly increased supernatant concentration of TNFα (*P* < 0.001; ANOVA; Fig. 2a), IL-6 (*P* < 0.001; ANOVA; Fig. 2b), and nitrite (*P* < 0.001; ANOVA; Fig. 2c) in WT BMDMs. In contrast, levels of each pro-inflammatory marker were significantly reduced in LPS/IL-4 stimulated NOX2^−/−^ BMDMs (*P* < 0.05 [TNFα, IL-6]; *P* < 0.001 [nitrite]; ANOVA; Fig. 2a–c). In addition, LPS/IL-4 significantly increased Arg1 protein (*P* < 0.001; ANOVA; Fig. 2d) and YM1 mRNA (*P* < 0.001; ANOVA; Fig. 2f) expression in WT BMDMs; expression of both anti-inflammatory markers were significantly increased beyond WT levels in NOX2^−/−^ cells (*P* < 0.01; ANOVA; Fig. 2d, f). CD206 is another anti-inflammatory activation marker, and LPS/IL-4 stimulation led to a significant decrease in CD206 mRNA expression in WT BMDMs (*P* < 0.05; ANOVA; Fig. 2g). In contrast, LPS/IL-4 significantly increased CD206 mRNA expression in NOX2^−/−^ BMDMs (*P* < 0.001; ANOVA; Fig. 2g).

### STAT1 activation is attenuated in NOX2^−/−^ BMDMs, whereas STAT6 activation is unchanged

**Fig. 3:**
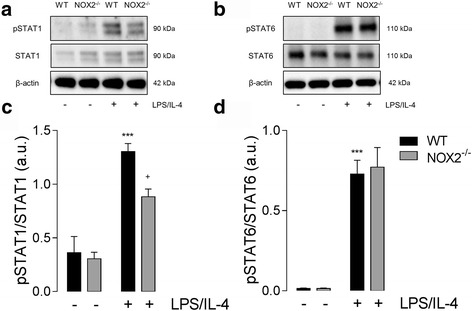
STAT1 activation is attenuated in NOX2^−/−^ BMDMs, whereas STAT6 activation is unchanged. (**a, b**) Protein expression of phosphorylated STAT1 and phosphorylated STAT6 in response to LPS/IL-4 stimulation was assessed by western immunoblotting. (**c**) LPS/IL-4 (both 10 ng/ml; 24 h) increased pSTAT1 expression (^**^ *P* ^*^ < 0.001, ANOVA) in WT BMDMs. In contrast, LPS/IL-4-induced increase in pSTAT1 was significantly reduced in NOX2^−/−^ BMDMs (^+^ *P* < 0.05, WT LPS/IL-4 vs. NOX2^−/−^ LPS/IL-4; ANOVA). (**d**) LPS/IL-4 significantly increased pSTAT6 (^***^ *P* < 0.001; ANOVA) in WT and NOX2^−/−^ BMDMs, but no genotype related differences were observed. All data are expressed as means (± SEM *n* = 5)

Having observed genotype-related changes in the expression of markers associated with the pro- and anti-inflammatory phenotypes, we set out to investigate changes in signaling pathways involved in the induction of these activation states. STAT1 is elevated following stimulation with pro-inflammatory cytokines and/or TLR agonists [5, 8]. Here, we demonstrate that STAT1 phosphorylation is significantly elevated in WT and NOX2^−/−^ BMDMs in response to LPS/IL-4 stimulation (*P* < 0.001; ANOVA; Fig. 3a, c). However, STAT1 activation was significantly reduced in LPS/IL-4 treated NOX2^−/−^ cells when compared to WT cells (*P* < 0.05; ANOVA; Fig. 3c). STAT6 is a part of the IL-4 signaling cascade [6, 33] and having observed enhanced expression of anti-inflammatory markers in NOX2^−/−^ cells we anticipated that these changes were mediated by STAT6 activation. LPS/IL-4 stimulation significantly increased pSTAT6 expression in WT and NOX2^−/−^ BMDMs (*P* < 0.001; ANOVA; Fig. 3b, d), however, there was no genotype-related difference in STAT6 activation.

### IL-10 and STAT3 signaling are increased in LPS/IL-4 stimulated NOX2^−/−^ BMDMs

**Fig. 4:**
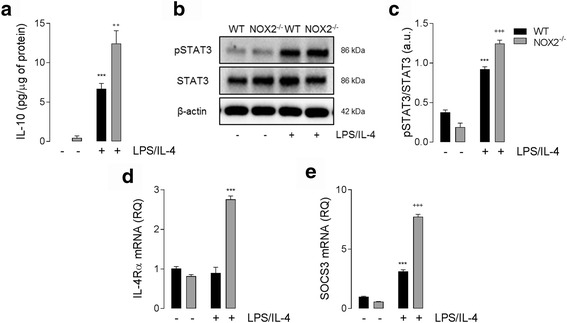
IL-10 production and downstream effectors are elevated in NOX2^−/−^ BMDMs in response to LPS/IL-4. (**a**) LPS/IL-4 (both 10 ng/ml; 24 h) stimulation significantly increased IL-10 protein concentration in WT and NOX2^−/−^ BMDMs (^***^ *P* < 0.001; ANOVA). The LPS/IL-4-induced increase in IL-10 concentration was significantly increased in NOX2^−/−^ BMDMs compared with WT BMDMs (^+++^p < 0.001, WT LPS/IL-4 vs. NOX2^−/−^ LPS/IL-4; ANOVA). (**b, c**) Protein expression of phosphorylated STAT3 was significantly increased in WT and NOX2^−/−^ BMDMs following LPS/IL-4 stimulation (^***^ *P* < 0.001; ANOVA; representative western immunoblot shown in **b**). LPS/IL-4-induced pSTAT3 expression was significantly increased in NOX2^−/−^ BMDMs compared with WT BMDMs (^+++^ *P* < 0.001, WT LPS/IL-4 vs. NOX2^−/−^ LPS/IL-4; ANOVA). (**d**) LPS/IL-4 significantly increased IL-4Rα mRNA expression NOX2^−/−^ BMDMs (^***^ *P* < 0.001; ANOVA). (**e**) SOCS3 mRNA expression was significantly increased in WT and NOX2^−/−^ BMDMs following LPS/IL-4 stimulation (^***^ *P* < 0.001; ANOVA). LPS/IL-4-induced SOCS3 expression was significantly increased NOX2^−/−^ BMDMs compared with WT BMDMs (^+++^ *P* < 0.001, WT LPS/IL-4 vs. NOX2^−/−^ LPS/IL-4; ANOVA). All data are expressed as means (± SEM, *n* = 6)

We demonstrated that the LPS/IL-4-mediated increase in anti-inflammatory markers in NOX2^−/−^ BMDMs was independent of STAT6 activation, therefore, we set out to determine what signaling pathways were involved in the NOX2-dependent shift towards an anti-inflammatory phenotype. We next measured supernatant concentration of IL-10 in LPS/IL-4-treated cells; there was a significant increase in IL-10 production in WT and NOX2^−/−^ BMDMs (*P* < 0.001; ANOVA; Fig. 4a), and this effect was greater in NOX2^−/−^ cells when compared to WT cells (*P* < 0.001; ANOVA; Fig. 4a). We also observed an increase in STAT3 phosphorylation in WT and NOX2^−/−^ cells (*P* < 0.001; ANOVA; Fig. 4b, c). Notably, STAT3 activation was significantly increased in LPS/IL-4 treated NOX2^−/−^ BMDMs when compared to WT BMDMs (*P* < 0.001; ANOVA; Fig. 4c). We then analyzed the expression of inflammatory markers downstream of IL-10 such as IL-4Rα and SOCS3 [34]. Consistent with the activation of IL-10 signaling there was a significant increase in IL-4Rα (*P* < 0.001; ANOVA; Fig. 4d) and SOCS3 (*P* < 0.001; ANOVA; Fig. 4e) mRNA expression in NOX2^−/−^ BMDMs when compared to levels in WT BMDMs.

### Increased anti-inflammatory activation in NOX2^−/−^ BMDMs is mediated through IL-10

**Fig. 5:**
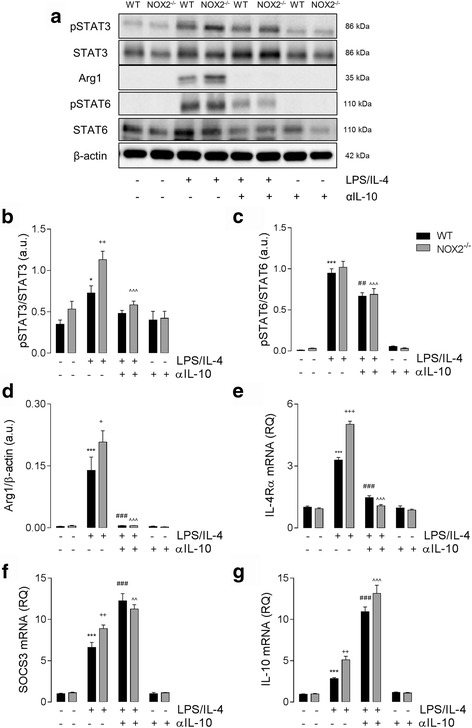
Increased anti-inflammatory activation in NOX2^−/−^ BMDMs is mediated through IL-10. (**a**) Representative immunoblots for pSTAT3, STAT3, Arg1, pSTAT6, STAT6, and β-actin. (**b**) LPS/IL-4 (both 10 ng/ml; 24 h) increased the expression of pSTAT3 in WT BMDMs (^*^ *P* < 0.05, vs. control; ANOVA), and LPS/IL-4-induced pSTAT3 expression was significantly increased in NOX2^−/−^ BMDMs (^++^ *P* < 0.01, WT LPS/IL-4 vs. NOX2^−/−^ LPS/IL-4; ANOVA). Co-incubation of BMDMs with neutralizing anti-IL-10 (αIL-10) attenuated the LPS/IL-4-induced increase in pSTAT3 in NOX2^−/−^ BMDMs (^^^^^ *P* < 0.001, NOX2^-/-^ LPS/IL-4 vs. NOX2^-/﻿-﻿﻿ ^LPS/IL-4 + αIL-10; ANOVA). (**c**) αIL-10 attenuated the LPS/IL-4-induced increase pSTAT6 expression (^***^ *P* < 0.001, control vs. LPS/IL-4; ^##^ *P* < 0.01, WT LPS/IL-4 vs. WT LPS/IL-4 + αIL-10; ^^^^^ *P* < 0.001, NOX2^−/−^ LPS/IL-4 vs. NOX2^−/−^ LPS/IL-4 + αIL-10; ANOVA), and no genotype-related changes were observed. (**d, e**) LPS/IL-4 induced an increase in Arg1 (**d**) protein expression, and IL-4Rα (**e**) mRNA expression in WT and NOX2^−/−^ BMDMs (^***^ *P* < 0.001, vs. control; ANOVA); these effects were significantly increased in NOX2^−/−^ BMDMs (^+^ *P* < 0.05, ^+++^ *P* < 0.001, WT LPS/IL-4 vs. NOX2^−/−^ LPS/IL-4; ANOVA). The LPS/IL-4 mediated effects on Arg1 and IL-4Rα expression were significantly reduced in the presence of αIL-10 (^^^^^p < 0.001, NOX2^−/−^ LPS/IL-4 vs. NOX2^−/−^ LPS/IL-4; ^###^p < 0.001, WT LPS/IL-4 vs. WT LPS/IL-4 + αIL-10; ANOVA). (**f, g**) SOCS3 and IL-10 mRNA expression was significantly increased in WT and NOX2^−/−^ BMDMs following LPS/IL-4 stimulation (^*^ *P* < 0.05, ^***^ *P* < 0.001, vs. control; ANOVA); the LPS/IL-4 effect was significantly increased in NOX2^−/−^ BMDMs compared with WT BMDMs (^*^ *P* < 0.05, WT LPS/IL-4 vs. NOX2^−/−^ LPS/IL-4; ANOVA). SOCS3 and IL-10 expression levels were significantly increased in the presence of αIL-10 (^^^^ *P* < 0.01, ^^^^^ *P* < 0.001, NOX2^−/−^ LPS/IL-4 vs. NOX2^−/−^ LPS/IL-4 + αIL-10; ^###^ *P* < 0.001, WT LPS/IL-4 vs. WT LPS/IL-4 + αIL-10; ANOVA). All data are expressed as means (± SEM, *n* = 6)

In order to test whether IL-10 mediates the anti-inflammatory shift in NOX2^−/−^ macrophages, WT and NOX2^−/−^ BMDMs were stimulated with LPS/IL-4 in the presence or absence of neutralizing αIL-10. As previously observed, LPS/IL-4 treatment significantly increased STAT3 activation in WT BMDMs (*P* < 0.01; ANOVA; Fig. 5a, b); an effect that was significantly increased in NOX2^−/−^ BMDMs (*P* < 0.05; ANOVA; Fig. 5b). In the presence of αIL-10, pSTAT3 expression was significantly reduced in NOX2^−/−^ BMDMs (*P* < 0.001; ANOVA; Fig. 5b), and the genotype effect was lost. Analysis of STAT6 activation confirmed equal pSTAT6 expression in WT and NOX2^−/−^ BMDMs following LPS/IL-4 stimulation (*P* < 0.001; ANOVA; Fig. 5a, c). IL-10 neutralization significantly decreased STAT6 activation in both genotypes (*P* < 0.01 [WT]; *P* < 0.001 [NOX2^−/−^]; ANOVA; Fig. 5c), but STAT6 activation remained significantly elevated when compared to control unstimulated cells. We next assessed Arg1 (Fig. 5a, d), IL-4Rα (Fig. 5e), SOCS3 (Fig. 5f), and IL-10 (Fig. 5g) expression in this experimental paradigm, and as predicted, there was a significant increase in each marker in NOX2^−/−^ BMDMs when compared to WT BMDMs (*P* < 0.05 [Arg1; SOCS3; IL-10]; *P* < 0.001 [IL-4Rα]; ANOVA; Fig. 5d–g). In the presence of αIL-10, the genotype effects on Arg1 and IL-4Rα expression were lost, and levels were returned to that of unstimulated cells (*P* < 0.001; ANOVA; Fig. 5d, e). LPS/IL-4-induced SOCS3 and IL-10 mRNA expression was increased in the presence of αIL-10 (*P* < 0.001, *P* < 0.01; ANOVA; Fig. 5f, g); however, the genotype-related effect on SOCS3 was lost when IL-10 was neutralized by αIL-10 (Fig. 5f).

### NOX2 deficiency results in increased cortical IL-10 expression following controlled cortical impact

**Fig. 6:**
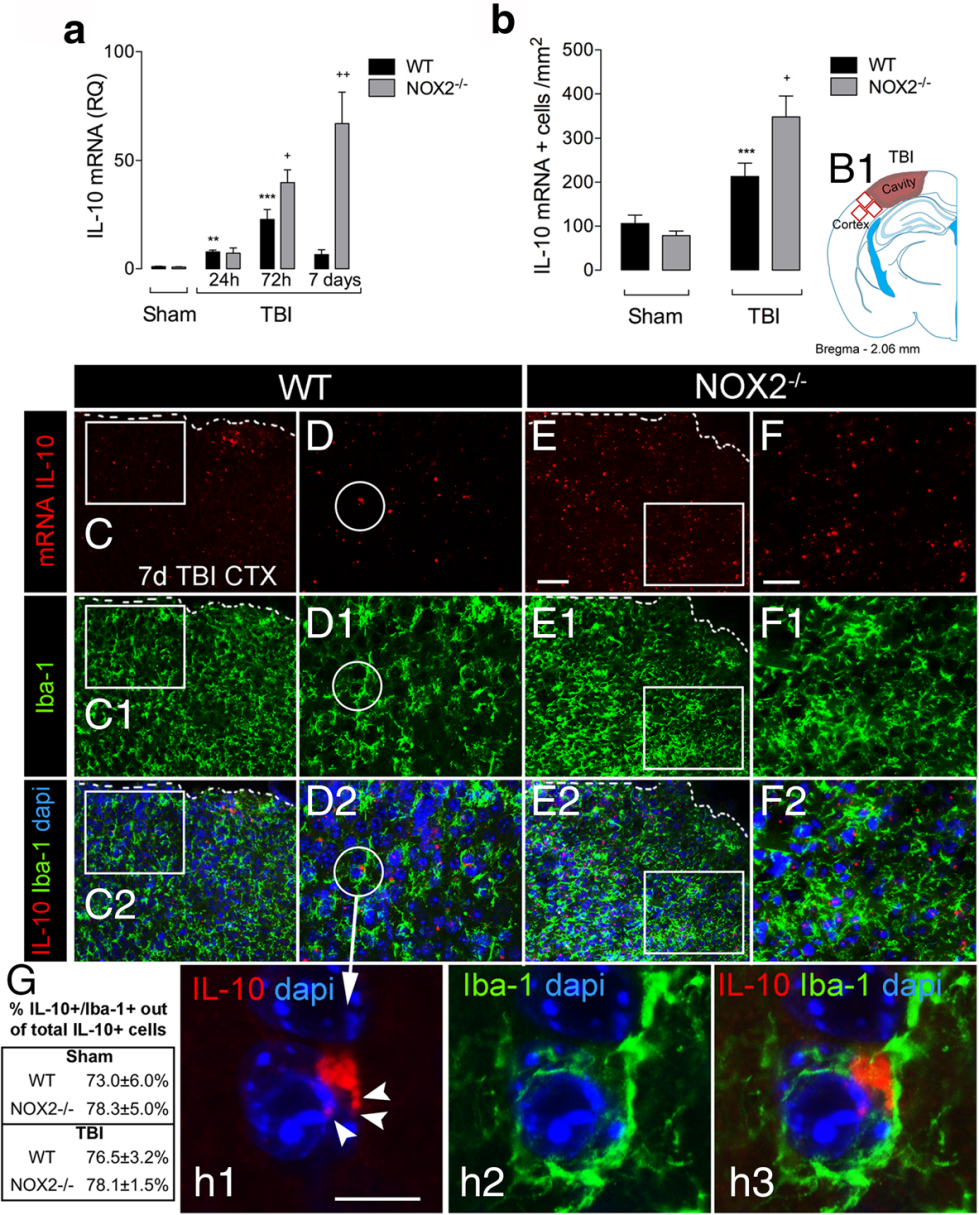
IL-10 is increased in the cortex of NOX2^−/−^ TBI mice. (**A**) IL-10 mRNA expression was assessed in the ipsilateral cortex of WT and NOX2^−/−^ sham and TBI mice at 24, and 72 h and 7 days post-injury. TBI increased IL-10 mRNA at all time points (^**^ *P* < 0.01, [24 h], ^***^ *P* < 0.001 [72 h], vs. sham; ANOVA). IL-10 expression was significantly increased in NOX2^−/−^ TBI mice at 72 h and 7 days (^+^ *P* < 0.05, ^++^\*P* < 0.01, WT TBI vs. NOX2^−/−^ TBI; ANOVA). All data are expressed as means (± SEM, *n*=5). (**B**) Quantification of IL-10 mRNA positive cells in the ipsilateral cortex of WT and NOX2^−/−^ sham and TBI mice at 7 days post-injury. TBI increased IL-10 expression when compared to sham (^***^ *P* < 0.001; ANOVA), and this effect was significantly greater in NOX2^−/−^ TBI mice (^+^ *P* < 0.05, WT TBI vs. NOX2^−/−^ TBI; ANOVA). All data are expressed as means (± SEM, *n* = 5). (**B1**) Schematic representation of the injured coronal brain section, the *red squares* indicate the fields that were examined in this study. (**C**–**H**) Representative images from the ipsilateral cortex of WT (**C**–**D2**) and NOX2^−/−^ (**E** *–* **F2**) TBI mice at 7 days post-injury. Images show reduced IL-10 positive cells (*red*) in the injured cortex of WT TBI (**C**, *inset* in **D**) compared to NOX2^−/−^ TBI mice (**E**, *inset* in **F**). IL-10 was predominantly expressed in microglia/macrophages detected by immunohistochemistry (*Iba-1*, *green*) and nuclei (*dapi*, *blue*) in both groups; WT (**C1** *–* **C2**, *insets* **in D** *–* **D2**) and in NOX2^−/−^ TBI mice (**E1** *–* **E2**, *insets* in **F2** *–* **F3**). A *white striped line* delineates the cavity and the perilesional cortex (**C**–**C2** and **E**–**E2**). (**G**) Table illustrating quantification analysis of %IL-10/Iba-1+ cells in the ipsilateral cortex of WT and NOX2^−/−^ sham and TBI mice. (**H**) High magnification (*inset circle* in **D**–**D2**) of a representative image for IL-10 positive (**h1**) microglia/macrophage (**h2**–**h3**, *Iba-1* in *green*, *dapi* in *blue*) showing cytoplasmic and perinuclear IL-10 mRNA expression with puncta spike that represents a single IL-10 mRNA transcript (*white arrowheads* in **h1**). *Scale bars* 75 μm for **C**–**C2** and **E**–**E2**; **F2**; 50 μm **D**–**D2**, **F**–**F2**; and 20 μm for **h1**–**h3**

Having demonstrated that IL-10/STAT3 signaling mediates the genotype-related changes in anti-inflammatory activation in NOX2^−/−^ BMDMs, we then set out to evaluate genotype related changes in IL-10 expression in the TBI brain using a moderate-level CCI model. IL-10 mRNA expression was measured in ipsilateral cortex of sham and TBI WT and NOX2^−/−^ mice at 24 and 72 h and 7 days post-injury (Fig. 6a). IL-10 mRNA expression was significantly increased in WT and NOX2^−/−^ TBI mice when compared to sham levels at all time points examined. Notably, there was a significant increase in IL-10 mRNA expression in NOX2^−/−^ TBI mice at 72 h and 7 days when compared to WT TBI mice (*P* < 0.05; [72 h]; *P* < 0.01 [7 days]; ANOVA; Fig. 6a). To identify the cellular source of IL-10, we combined IL-10 mRNA FISH with immunohistochemical markers for macrophages/microglia in the ipsilateral cortex of WT and NOX2^−/−^ sham and TBI mice at 7 days post-injury. There was a significant increase in IL-10-positive cells in the cortex of NOX2^−/−^ TBI mice when compared to WT TBI mice (*P* < 0.05; ANOVA; Fig. 6b), and approximately 78% of the IL-10-positive cells were Iba-1-positive macrophages/microglia (representative images Fig. 6c–h). Together, these data indicate that NOX2 deficiency results in a significant increase in IL-10 expression in macrophages/microglia during the acute-subacute phase following TBI.

### Increased anti-inflammatory marker expression in the cortex of NOX2^−/−^ TBI mice is associated with enhanced IL-10/STAT3 signaling

**Fig. 7:**
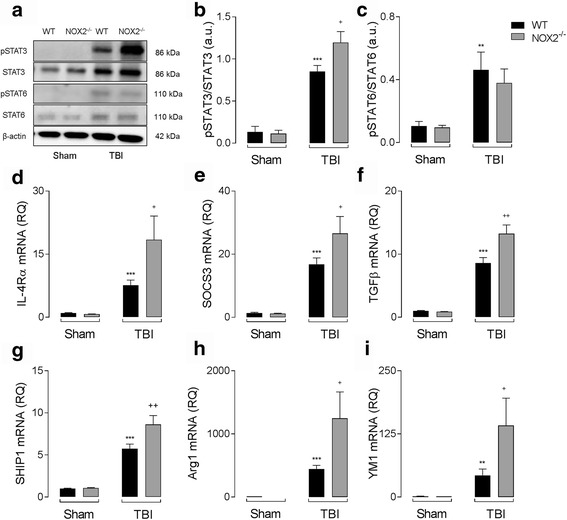
Increased expression of anti-inflammatory markers in the cortex of NOX2^−/−^ TBI mice is associated with enhanced IL-10/STAT3 signaling. (**a**) Protein expression of phosphorylated STAT3 and phosphorylated STAT6 was assessed by western immunoblotting in the ipsilateral cortex of WT and NOX2^−/−^ sham and TBI mice at 72 h post-injury. (**b**) pSTAT3 expression was significantly increased in WT and NOX2^−/−^ TBI mice (^***^ *P* < 0.001, vs. sham; ANOVA). pSTAT3 expression was significantly increased in the cortex of NOX2^−/−^ TBI mice when compared to WT TBI mice (^+^ *P* < 0.05, WT TBI vs. NOX2^−/−^ TBI; ANOVA). (**c**) TBI significantly increased pSTAT6 expression in the cortex of WT and NOX2^−/−^ mice (^***^ *P* < 0.001, vs. sham; ANOVA); there was no genotype-related differences in pSTAT6 expression following TBI. (**d-i**) TBI significantly increased IL-4Rα (**d**), SOCS3 (**e**), TGFβ (**f**), SHIP1 (**g**), Arg1 (**h**), and YM1 (**i**) mRNA expression in the ipsilateral cortex of WT and NOX2^−/−^mice at 72 h post-injury (^***^ *P* < 0.001, vs. sham; ANOVA). The expression of each anti-inflammatory marker was significantly increased in NOX2^−/−^ TBI mice when compared to WT TBI mice (^+^ *P* < 0.05, ^++^ *P* < 0.01, WT TBI vs. NOX2^−/−^ TBI; ANOVA; **d-i**). All data are expressed as means (±SEM; *n* = 5)

IL-10 expression was enhanced in NOX2^−/−^ TBI mice compared to WT TBI mice; therefore, we next assessed STAT3 and STAT6 signaling pathways and anti-inflammatory marker expression at 72 h post-injury. TBI significantly increased the expression of pSTAT3 in the ipsilateral cortex of both WT and NOX2^−/−^ mice (*P* < 0.001; ANOVA; Fig. 7b). Notably, STAT3 activation was robustly and significantly increased in NOX2^−/−^ TBI mice when compared to WT TBI mice (*P* < 0.05; ANOVA; Fig. 7b). STAT6 activation was increased in WT and NOX2^−/−^ TBI mice (*P* < 0.01; ANOVA; Fig. 7c); however, there was no genotype-related change in pSTAT6 expression. In addition, TBI resulted in a significant increase in the cortical expression of IL-4Rα (*P* < 0.001; ANOVA; Fig. 7d), SOCS3 (*P* < 0.001; ANOVA; Fig. 7e), TGFβ (*P* < 0.001; ANOVA; Fig. 7f), SHIP1 (*P* < 0.001; ANOVA; Fig. 7g), Arg1 (*P* < 0.001; ANOVA; Fig. 7h), and YM1 (*P* < 0.01; ANOVA; Fig. 7i) mRNA in WT and NOX2^−/−^ TBI mice. The injury effect on inflammatory markers downstream of IL-10 was significantly increased in the ipsilateral cortex of NOX2^−/−^ TBI mice when compared to WT TBI mice (*P* < 0.05 [IL-4Rα; SOCS3; Arg1; YM1]; *P* < 0.001 [SHIP1; TGFβ]; ANOVA; Fig. 7**d–i**; also Additional file 1: Table S1).

### IL-10 promotes an anti-inflammatory environment in NOX2^−/−^ mice following TBI

**Fig. 8:**
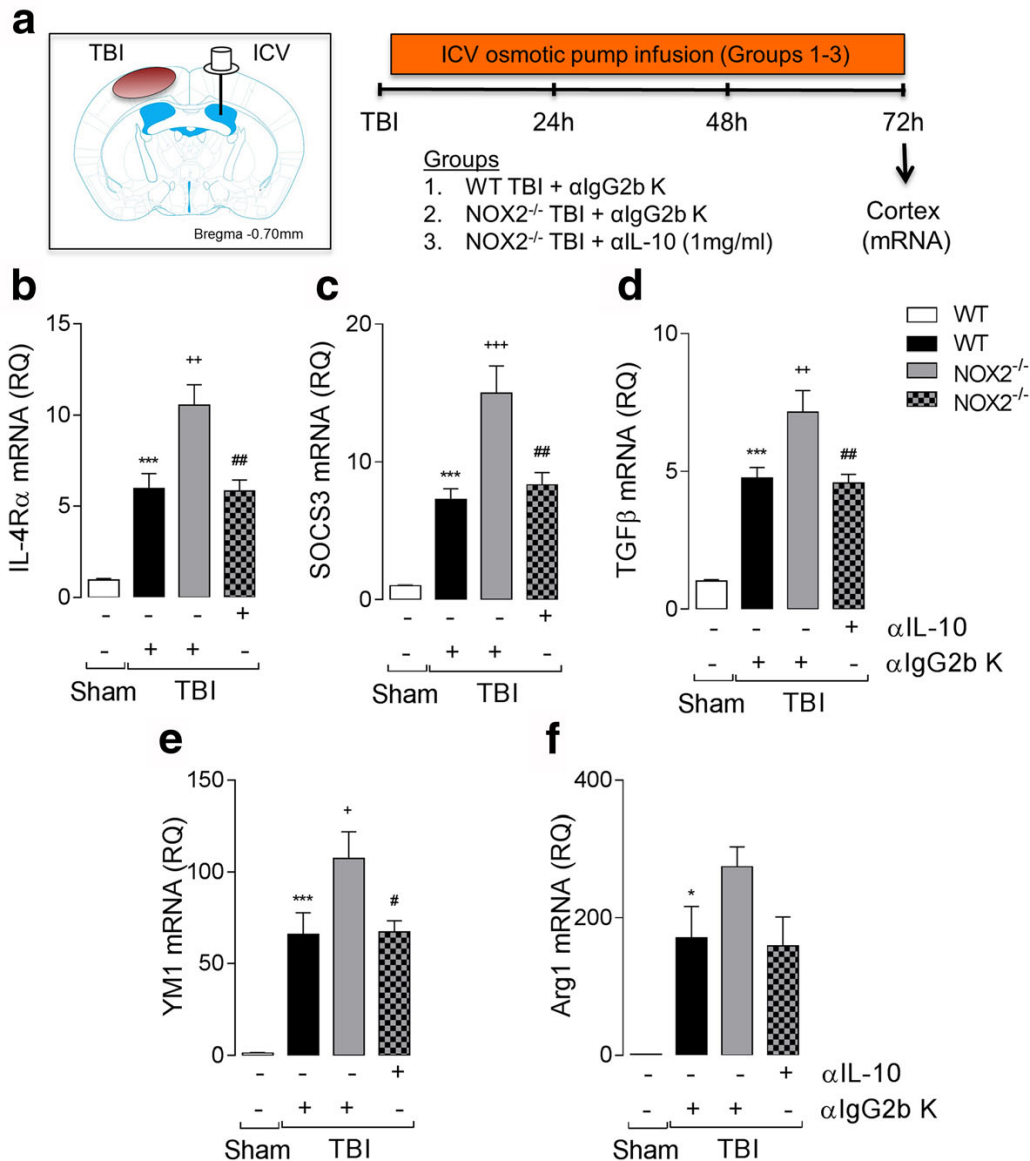
IL-10 promotes an anti-inflammatory environment in cortex of NOX2^−/−^ TBI mice. (**a**) Neutralizing anti-IL-10 (αIL-10; 1 mg/ml, i.c.v.) or control αIgG2b K was administered to WT or NOX2^−/−^ TBI mice for 72 h, and ipsilateral cortex tissue was collected for mRNA analysis. (**b-f**) TBI increased IL-4Rα (**b**), SOCS3 (**c**), TGFβ (**d**), YM1 (**e**), and Arg1 (**f**) mRNA expression in the ipsilateral cortex of WT and NOX2^−/−^mice (^***^ *P* < 0.001, vs. sham; ANOVA). TBI effect on anti-inflammatory marker expression was significantly increased in NOX2^−/−^ TBI mice when compared to WT TBI mice (^+^ *P* < 0.05, ^++^ *P* < 0.01, ^+++^ *P* < 0.001, WT αIgG2b K TBI vs. NOX2^−/−^ αIgG2b K TBI; ANOVA). αIL-10 treatment significantly reduced the expression of all anti-inflammatory markers in NOX2^−/−^ TBI mice (^#^ *P* < 0.05, ^##^ *P* < 0.01, NOX2^−/−^ αIgG2b K TBI vs. NOX2^−/−^ αIL-10 TBI; ANOVA). All data are expressed as means (±SEM; *n* = 6)

Finally, to determine if IL-10 promotes the anti-inflammatory response in NOX2^−/−^ TBI mice, we blocked IL-10 in the brain using the IL-10 neutralization strategy that we employed in prior BMDM studies. To achieve this, WT mice and NOX2^−/−^ TBI mice had an i.c.v. osmotic pump implanted on the contralateral side to infuse neutralizing αIL-10 (1 mg/ml) or equal concentration isotype control αIgG (αIgG2b K) into the lateral ventricle for 72 h, after which ipsilateral cortex tissue was collected to assess anti-inflammatory markers (Fig. 8a). TBI increased the expression of IL-4Rα (*P* < 0.001; ANOVA; Fig. 8b), SOCS3 (*P* < 0.001; ANOVA; Fig. 8c), TGFβ (*P* < 0.001; ANOVA; Fig. 8d), YM1 (*P* < 0.001; ANOVA; Fig. 8e), and Arg1 (*P* < 0.05; ANOVA; Fig. 8f) mRNA in all TBI mice compared to WT sham mice. As predicted, the effect of TBI on IL-4Rα, SOCS3, TGFβ, and YM1 was significantly increased in NOX2^−/−^ TBI mice compared to WT TBI mice (*P* < 0.05 [YM1]; *P* < 0.01 [IL-4Rα; TGFβ]; *P* < 0.001 [SOCS3]; ANOVA; Fig. 8b–e). Notably, the NOX2^−/−^-dependent upregulation of anti-inflammatory markers after TBI was significantly reduced when IL-10 was neutralized using αIL-10, such that the αIL-10-treated NOX2^−/−^ TBI group returned to αIgG-treated WT TBI levels (*P* < 0.05 [YM1]; *P* < 0.01 [IL-4Rα; SOCS3; TGFβ]; ANOVA; Fig. 8b–e).

## Discussion

Redox signaling regulates macrophage responses during inflammation [16], and NOX2/ROS are implicated in secondary neuroinflammatory cascades in ischemic brain injury [35, 36], spinal cord injury (SCI) [37, 38], and TBI [21, 23, 26]. NOX2 is highly expressed in macrophage/microglia up to 12 months after moderate-to-severe TBI [21], and inhibition of NOX2 is neuroprotective and reduces inflammatory-mediated neuronal cell death after TBI [23–26]. We recently reported that NOX2 inhibition also enhances anti-inflammatory activation of macrophage/microglia following TBI [24]. Thus, NOX2 may act as a critical switch between pro- and anti-inflammatory phenotypes after TBI. Similar findings in SCI [37, 39] and stroke [40] models indicate that this may be a common cellular mechanism that regulates CNS injury responses. Therefore, the goal of the present study was to investigate signaling pathways that regulate NOX2-dependent phenotypic changes in macrophages and relate these signaling events back to TBI. To this end, we developed an in vitro model of macrophage activation that mimics characteristic features of the mixed pro- and anti-inflammatory phenotypes observed in the TBI brain [12–14], and used it to identify IL-10-STAT3 signaling as a key mechanism by which NOX2 inhibition regulates macrophage activation states after TBI.

Whereas many previous studies have demonstrated that macrophages/microglia in vitro can be stimulated to adopt specific phenotypes, it appears less likely that such defined phenotypes exist in vivo [9]. We, and others, have demonstrated that following brain trauma macrophages/microglia adopt mixed phenotypes and express both pro and anti-inflammatory activation markers [12–14]. Here, we demonstrated that similar to the activation profile in the TBI brain, macrophages stimulated with combined LPS/IL-4, but not LPS or IL-4 alone, upregulated mixed phenotypic markers associated with both pro- and anti-inflammatory activation states. When we compared the responses of WT and NOX2^−/−^ BMDMs to the mixed LPS/IL-4 stimulation model, we found that NOX2 deficiency was associated with decreased production of pro-inflammatory mediators. This activation profile is consistent with prior studies in NOX2^−/−^ microglia exposed to LPS challenge [27, 41]. There was also a significant increase in anti-inflammatory activation markers in NOX2^−/−^ BMDMs. These findings are significant because there are increased numbers of anti-inflammatory microglia/macrophages in the injured cortex of NOX2^−/−^ TBI mice [24]. Of note, it was not possible to promote this NOX2-dependent phenotype shift when BMDMs were treated with only pro-inflammatory stimuli (LPS or IFNγ alone; data not shown) or anti-inflammatory stimuli (IL-4 alone; data not shown), highlighting the importance of mixed environmental stimuli (combined LPS/IL-4) for NOX2-dependent macrophage phenotype responses. In the context of TBI, it is intriguing to speculate on the significance of these stimulus-driven effects such that they highlight intrinsic tension between activation pathways. Similar to responses in wound healing macrophages [42], coincident pro- and anti-inflammatory activation may be an attempt to fine-tune the injury response in which the two pathways support each other to facilitate neuroprotection and immune resolution that may be important for neurorestoration after TBI, thereby revealing their complementary nature. However, complete absence of a molecular signaling cascade (activation by only pro- (LPS) or anti-inflammatory (IL-4) stimuli in BMDMs) or the loss of molecular signaling over time (dominant NOX2 response after TBI [12]) may significantly impact the functional effects of the opposite microglial/macrophage activation state.

We investigated the impact of NOX2 deficiency on signaling cascades involved in regulating macrophage phenotypes, with particular emphasis on mechanisms that drive anti-inflammatory activation that may be relevant for neuroprotection and neurorestoration following TBI. The role of STAT1 in the induction of the pro-inflammatory phenotype is well-established [43], and STAT1 is required for LPS-induced gene expression in macrophages [5]. In our model, the mixed LPS/IL-4 stimulus significantly increased pSTAT1 expression in WT and NOX2^−/−^ BMDMs, but STAT1 activation was significantly reduced in NOX2^−/−^ BMDMs. This is in agreement with a previous report where NOX deficient macrophages incubated in the presence of IFNγ/LPS exhibited decreased STAT1 activation [44]. In addition, previous work demonstrated that LPS-induced IFNβ is required for STAT1 activation [45], and this may be a mechanism that explains reduced pSTAT1 and decreased pro-inflammatory marker expression in NOX2^−/−^ BMDMs.

IL-4 stimulation increases the expression of markers associated with the anti-inflammatory phenotype, and STAT6 activation appears to be essential for driving these changes [6]. Our recent study suggested that the enhanced anti-inflammatory environment in the cortex of NOX2^−/−^ TBI mice is mediated, at least in part, by IL-4Rα-positive infiltrating macrophages [24]. Here, we demonstrated that LPS/IL-4 treatment increased anti-inflammatory markers in NOX2^−/−^ BMDMs when compared to WT BMDMs. Unexpectedly, we observed no genotype-related change in STAT6 activation, which conflicts with a prior report showing that pSTAT6 expression is enhanced in NOX2^−/−^ BMDMs [44]. Such discrepancies may be due to the different activation stimuli employed in each study. Nevertheless, the results from the current study suggest that enhanced anti-inflammatory changes in NOX2^−/−^ BMDMs are not mediated by increased STAT6 signaling.

Having established that anti-inflammatory responses in NOX2^−/−^ BMDMs were not due to enhanced STAT6 activation, we then identified an alternative pathway that could mediate these effects. IL-10 is a pleiotropic immunoregulatory cytokine that plays a critical role in controlling inflammation and immune responses [46]. IL-10 regulates TLR signaling [47], and IL-10 deficient mice exhibit exaggerated responses to LPS [48]. IL-10 can also enhance anti-inflammatory activation induced by IL-4 [7]. Here, we show that LPS/IL-4 stimulation enhances IL-10 release, an effect which is robustly increased in NOX2^−/−^ BMDMs. These data are consistent with a recent report showing that ROS inhibition using a mitochondrial complex 1 inhibitor decreases LPS-induced activation of macrophages and increases IL-10 production [49]. IL-10 mediates its effects through activation of STAT3 [50], and in the current study we found that STAT3 signaling was significantly increased in NOX2^−/−^ BMDMs. Stimulation of macrophages with IL-10 leads to the upregulation of several genes, including IL-4Rα and SOCS3 [34], and in response to LPS/IL-4, mRNA expression of both markers was significantly increased in NOX2^−/−^ compared to WT BMDMs.

To investigate whether IL-10 plays a critical role in the NOX2-dependent phenotype switch, WT and NOX2^−/−^ BMDMs were stimulated with LPS/IL-4 in the presence or absence of a neutralizing αIL-10 antibody. Increased pSTAT3 expression in NOX2^−/−^ BMDMs was attenuated in the presence of αIL-10, demonstrating that the genotype-related effect on STAT3 activation was mediated by IL-10. IL-4-stimulated macrophage activation can be synergized by IL-10 [7], and when IL-10 was neutralized, there was a significant reduction in IL-4Rα and Arg1. Interestingly, when IL-10 was neutralized, the LPS/IL-4 effects on SOCS3 and IL-10 were further increased; significantly, the NOX2 genotype-related effects were abolished with αIL-10 treatment. These observations are consistent with the fact that TLR4 activation induces SOCS3 and IL-10 expression [32, 51]; by removing the inhibitory effect of IL-10, LPS-induced changes are increased. Together, these data demonstrate that in the absence of NOX2, there is increased IL-10/STAT3 signaling, which in turn results in enhanced anti-inflammatory macrophage activation.

Clinically, IL-10 is upregulated during the acute phase after moderate-to-severe TBI [52, 53], and several pre-clinical studies report neuroprotective effects of IL-10 treatment in acute brain injury that can be attributed to its anti-inflammatory activities [54–56]. IL-10 treatment produces strong neuroprotective effects in LPS-induced dopaminergic neurotoxicity models [57], and these effects are mediated by inhibition of microglial activation and NOX2-dependent ROS in neuron-glia co-cultures. In the present study, we demonstrated that IL-10 expression was increased in the ipsilateral cortex of WT and NOX2^−/−^ TBI mice, and that this effect was significantly greater in NOX2^−/−^ animals. Importantly, our in situ analysis showed that IL-10 expression was robustly upregulated in approximately 78% of macrophages/microglia within the injured cortex of NOX2^−/−^ TBI mice. We previously identified the peak of macrophage infiltration after TBI to be at 72 h post-injury [12], and this is the same time point at which IL-10 expression is maximal in WT mice. There was also increased pSTAT3 expression, but no change in STAT6 activation, in TBI NOX2^−/−^ mice at 72 h post-injury, suggesting that the changes observed were as a result of increased STAT3 activity. STAT3 activation has been described in secondary injury responses following TBI and SCI [58–60]. Although some studies have suggested a beneficial role [59], others have reported that STAT3 activation is detrimental [61]. These conflicting reports highlight the complexity of STAT3 activation in secondary injury and differential STAT3 cellular responses during acute CNS injury (STAT3 effects in glia vs. neurons). Here, we clearly demonstrate that NOX2 deficiency resulted in increased STAT3 activation in macrophages (BMDMs), and that a similar STAT3 activation profile was observed in the injured cortex of NOX2^−/−^ TBI mice. In addition to enhanced STAT3 signaling, several genes downstream of IL-10 (IL-4Rα, TGFβ, SOCS3, SHIP1) and other anti-inflammatory genes (Arg1 and YM1) were robustly upregulated in the injured cortex of NOX2^−/−^ TBI mice. Therefore, despite no differences in IL-4-mediated signaling, our data indicate that the anti-inflammatory response in NOX2^−/−^ TBI mice may be as a result of enhanced IL-10/STAT3 signaling.

Finally, to address whether IL-10 is essential for the enhanced anti-inflammatory environment in NOX2^−/−^ TBI mice, we used a neutralizing antibody to inhibit IL-10 within the CNS as previously described [29]. After IL-10 neutralization, the TBI-induced increase in expression of anti-inflammatory markers in NOX2^−/−^ TBI mice was significantly reduced compared to expression levels in IgG control NOX2^−/−^ TBI mice. These results indicate that IL-10 is a major contributor to the anti-inflammatory environment in the injured cortex of NOX2^−/−^ TBI mice. When combined with our recent report [24], these studies indicate that NOX2 inhibition in macrophages switches them towards an anti-inflammatory phenotype through an IL-10/STAT3 dependent pathway, which may contribute to the neuroprotective effects that result in long-term neurological recovery after TBI. Interestingly, following SCI infiltrating monocyte-derived macrophages that display an immunoregulatory phenotype with high IL-10 expression play a critical role in controlling the inflammatory response within the lesion microenvironment, which in turn is associated with improved motor function recovery [62]. Furthermore, adoptive transfer of anti-inflammatory macrophages that infiltrate the lesion site after SCI where they produce high levels of IL-10 and IL-13 to promote axon remyelination and preserve neuronal function [63].

## Conclusions

**Fig. 9:**
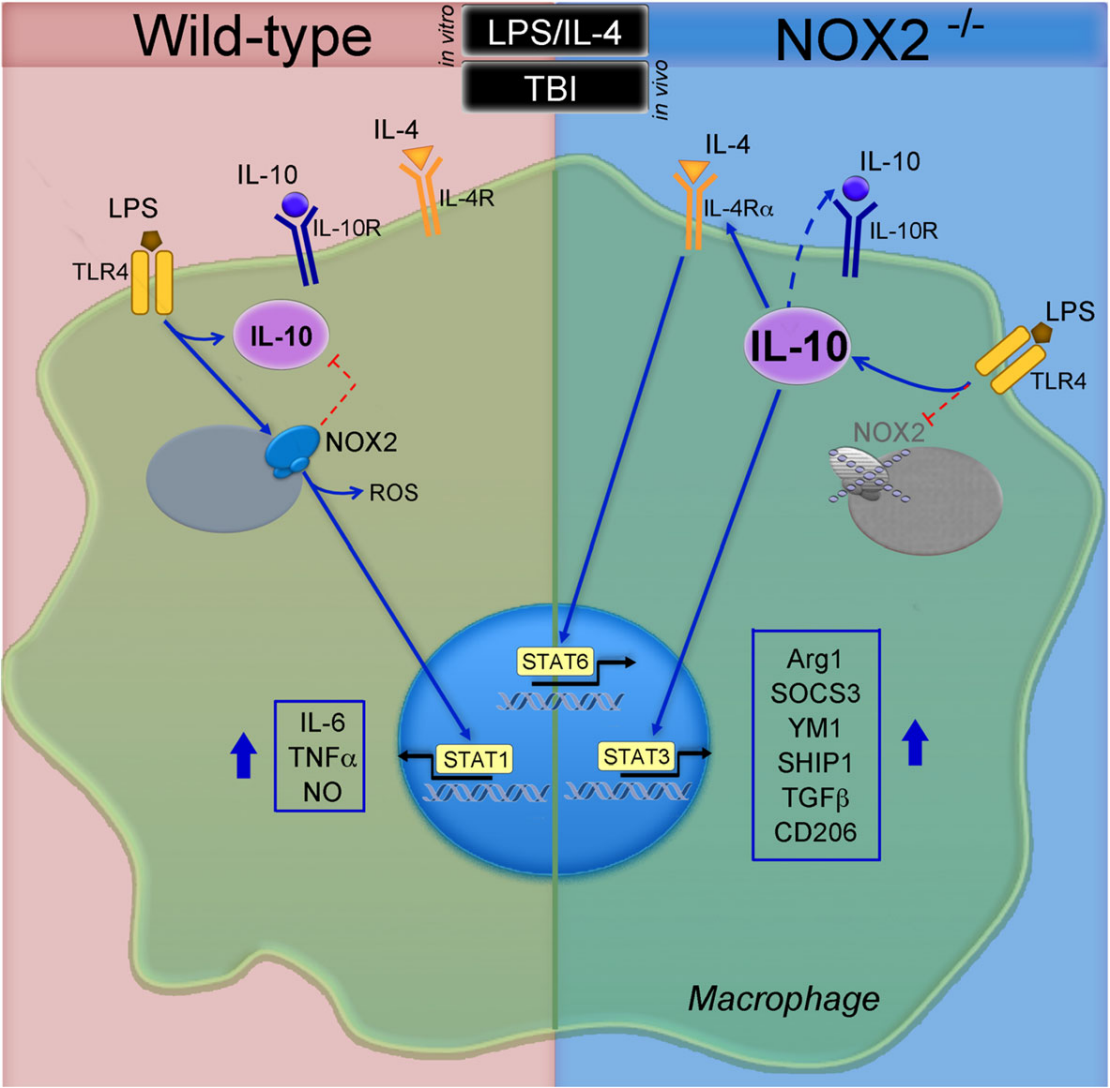
Schematic of NOX2 regulation of macrophage activation. Activation of TLR4 initiates a signaling cascade that leads to activation of NOX2 and increased ROS production; this in turn induces the activation of STAT1, which leads to an increase in the production of pro-inflammatory mediators. TLR4 signaling also induces the production of IL-10, which acts in autocrine fashion to regulate TLR4-induced changes. In NOX2^−/−^ cells, TLR4-induced changes are significantly different; NOX2-deficiency is associated with a decrease in pro-inflammatory markers and significantly greater IL-10 production. This increase in IL-10 leads to a robust increase in STAT3 signaling, which in conjunction with STAT6 activation, leads to increased expression of anti-inflammatory markers

These studies demonstrate that in response to mixed inflammatory stimuli BMDMs from NOX2^−/−^ mice exhibit decreased pro-inflammatory activation, and a concomitant increase in anti-inflammatory activation, and that this switch is IL-10/STAT3-dependent (Fig. 9). Furthermore, our in vivo TBI studies indicate that NOX2 deficiency drives an anti-inflammatory response in the injured cortex that is mediated by IL-10. Overall, these findings indicate that interventions that inhibit NOX2 activity in macrophages/microglia may improve outcomes following TBI not only by limiting pro-inflammatory and neurotoxic responses, but also by enhancing IL-10-mediated responses that are neuroprotective.

## Additional file

Additional file 1: Table S1. Average CT values of genes measured by qPCR in the cortex of SHAM and TBI (72 h). (TIF 126 kb)

## Abbreviations

ANOVA: Analysis of variance
Arg1: Arginase-1
BMDM: Bone marrow-derived macrophage
CCI: Controlled cortical impact
CD206: Mannose receptor
FISH: Fluorescent In Situ Hybridization
i.c.v.: Intracerebroventricular
IFN-γ: Interferon-γ
IGF-1: Insulin-stimulating growth factor-1
IL-10: Interleukin-10
IL-4: Interleukin-4
IL-6: Interleukin-6
LPS: Lipopolysaccharide
NO: Nitric oxide
NOS2: Nitric oxide synthase
NOX2: NADPH Oxidase
NOX2^−/−^: NOX2 deficient
ROS: Reactive oxygen species
SCI: Spinal cord injury
SOCS: Suppressor of cytokine signaling
STAT: Signal transducer activator of transcription
TBI: Traumatic brain injury
TLR: Toll-like receptors
TNF: Tumor necrosis factor
