# Supplementary figures and images for: NOX2 deficiency alters macrophage phenotype through an IL-10/STAT3 dependent mechanism: implications for traumatic brain injury
Source: J Neuroinflammation. 2017 Mar 24;14:65. doi: 10.1186/s12974-017-0843-4 (PMC5366128; doi:10.1186/s12974-017-0843-4)

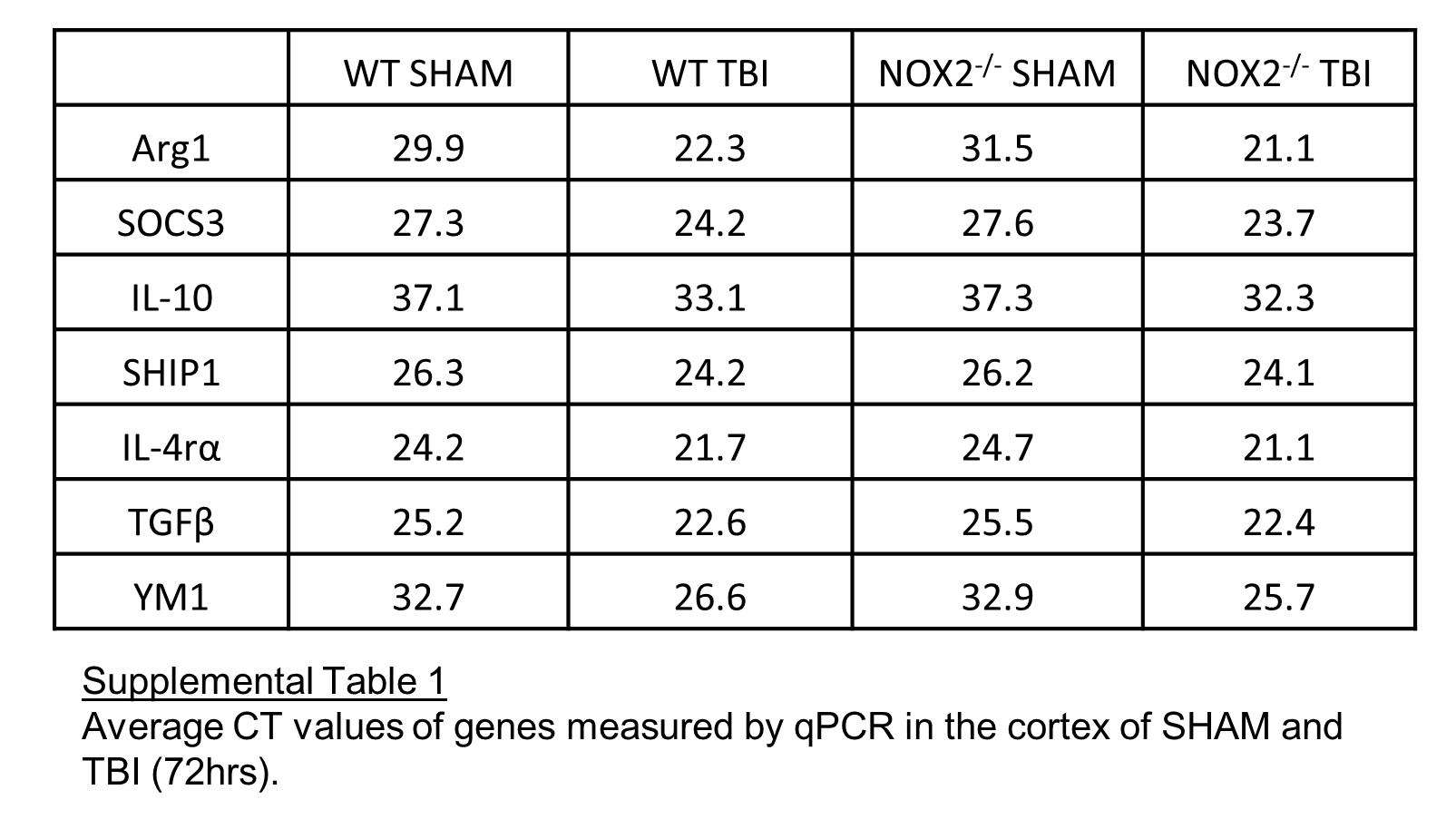

Supplement: Additional file 1: Table S1. — Average CT values of genes measured by qPCR in the cortex of SHAM and TBI (72 h). (TIF 126 kb) [file 12974_2017_843_MOESM1_ESM.tif]
